# Supplementary material for: Design of experiments for fine-mapping quantitative trait loci in livestock populations
Source: BMC Genet. 2020 Jun 29;21:66. doi: 10.1186/s12863-020-00871-1 (PMC7324978; doi:10.1186/s12863-020-00871-1)
Supplement: Supplementary file 1 — Additional file 1 PDF file containing additional figures. [file 12863_2020_871_MOESM1_ESM.pdf]

Additional Figures

Wittenburg *et al.* "Design of Experiments for Fine-Mapping Quantitative Trait Loci in Livestock Populations" *BMC Genetics*

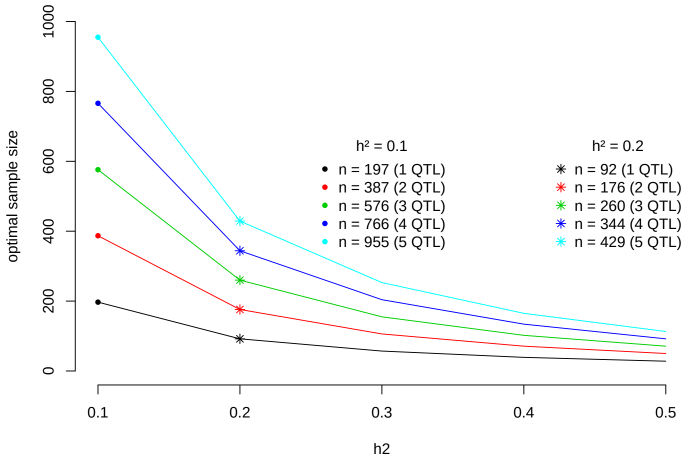

Figure S.1 Optimal sample size estimated from the single-SNP model and depending on heritability. Pointwise type-I error was corrected using the simple$\mathcal{M}$ method.

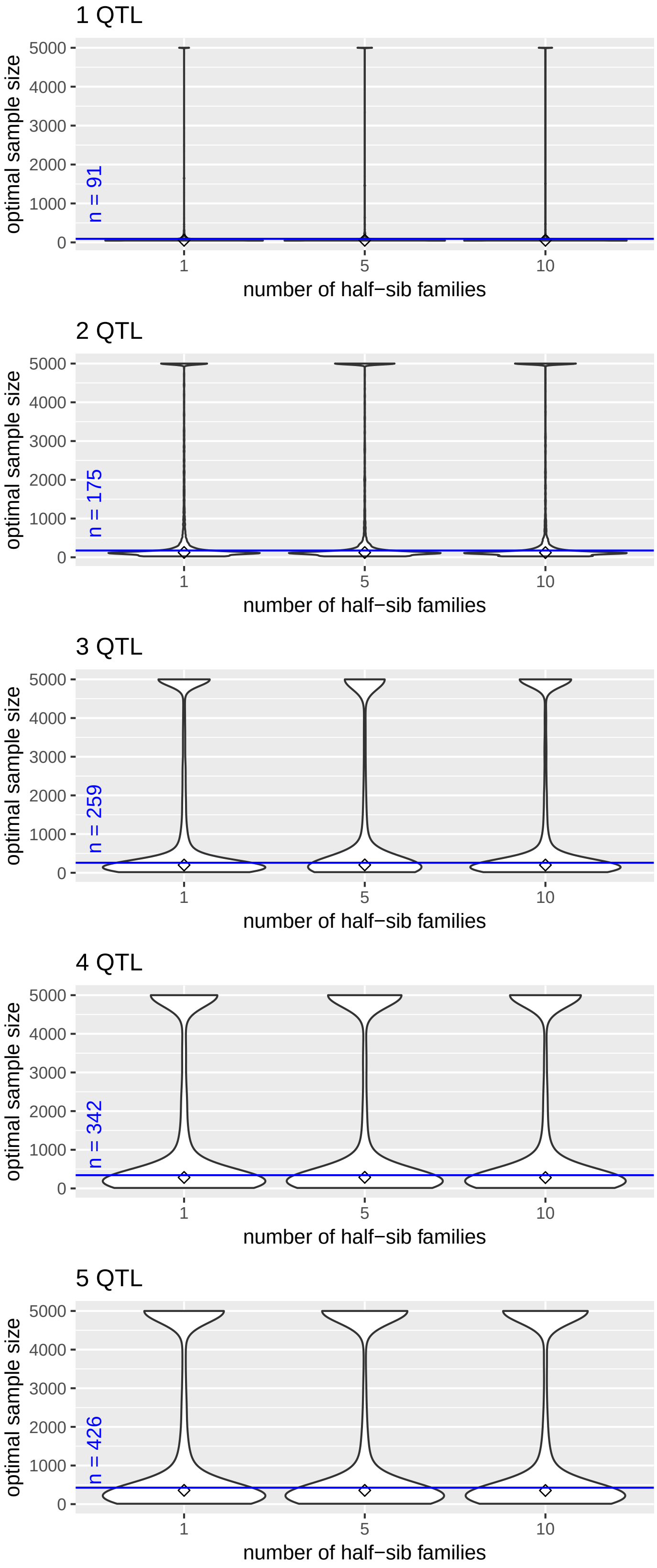

Figure S.2 Distribution of optimal sample size. Violinplot of n_{opt} vs. number of half-sib families for different numbers of QTL signals in a multi-SNP model. The parent generation was simulated 100 times and 100 random draws of positions of QTL signals were analyzed in each run, $h^2 = 0.2$. The diamond indicates the median of n_{opt} and the blue line marks the results based on a single-SNP model.

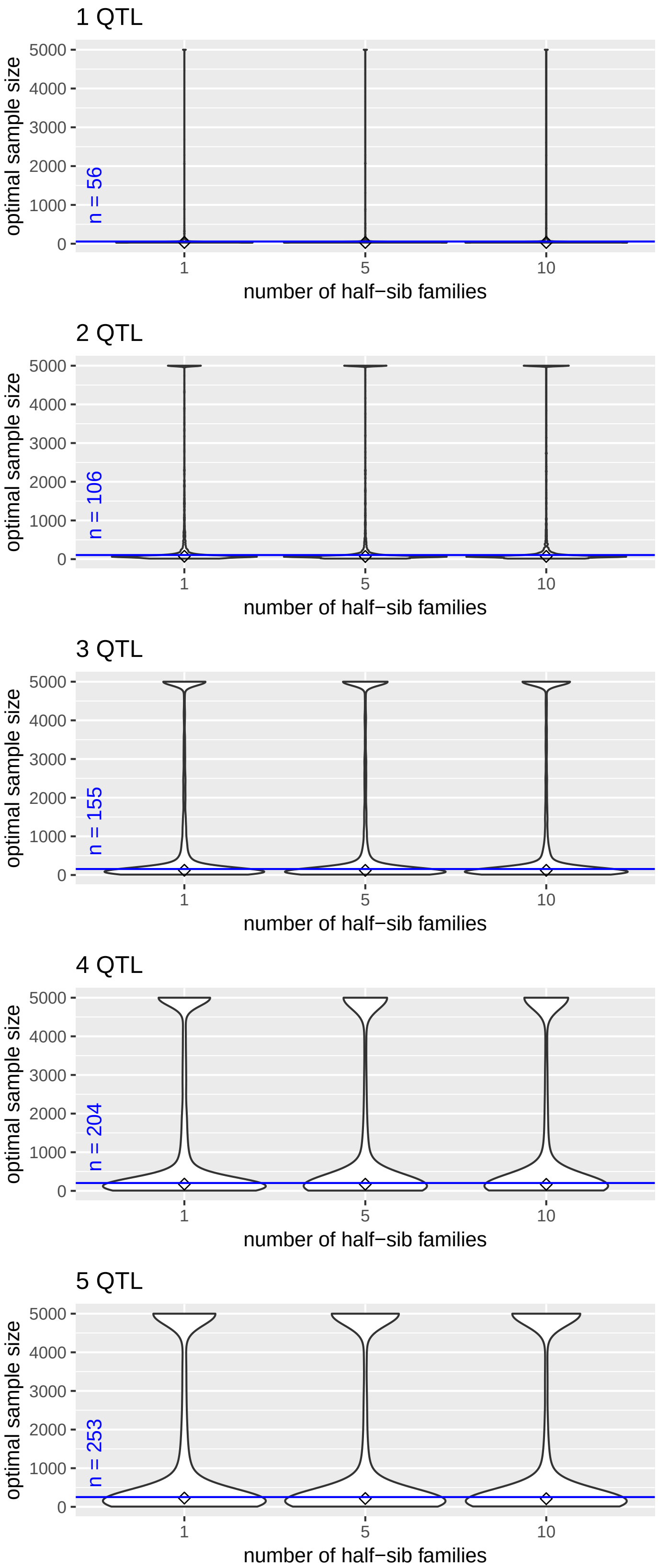

Figure S.3 Distribution of optimal sample size. Violinplot of n_{opt} vs. number of half-sib families for different numbers of QTL signals in a multi-SNP model. The parent generation was simulated 100 times and 100 random draws of positions of QTL signals were analyzed in each run, $h^2 = 0.3$. The diamond indicates the median of n_{opt} and the blue line marks the results based on a single-SNP model.

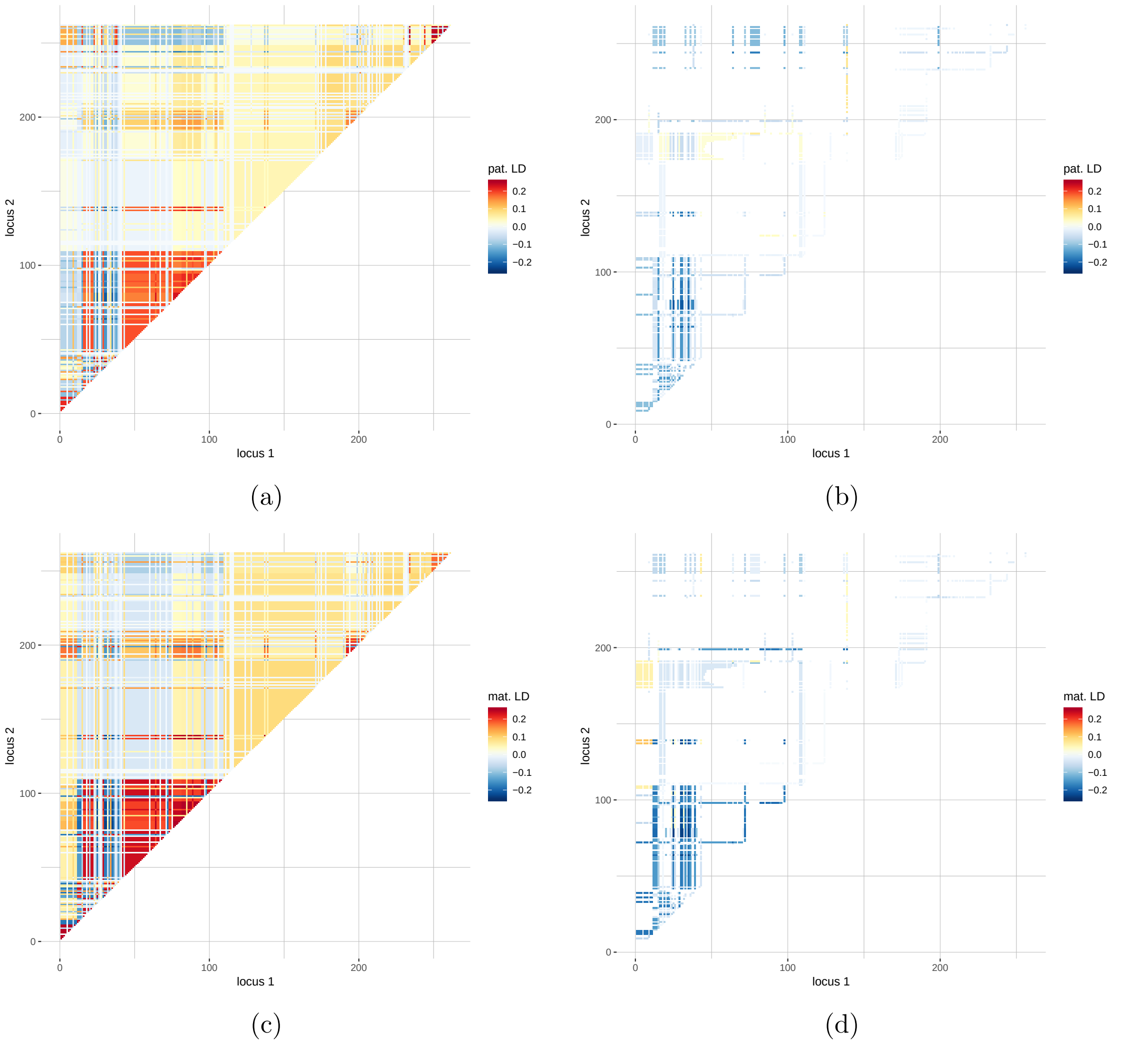

Figure S.4 Separation of dependence between SNPs in a single simulated data set with $N = 10$ sires. (a) Paternal covariance, (b) entries selected from paternal covariance which belong to 10 % highest sample size ($n_{\text{opt}} \geq 864$), (c) maternal covariance, (d) entries selected from maternal covariance which belong to 10 % highest sample size. All possible SNP pairs were evaluated to detect two QTL signals ($h^2 = 0.1$).

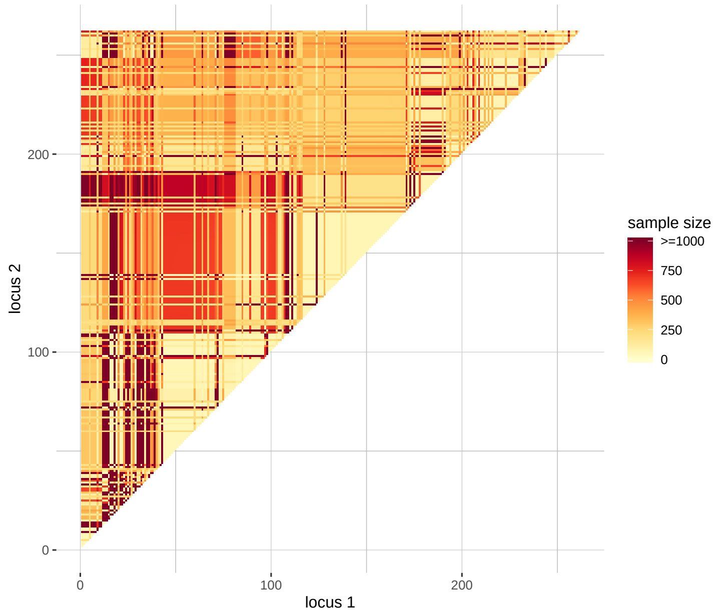

Figure S.5 Relationship of optimal sample size with distance between QTL signals in a single simulated data set with $N = 10$ sires. All possible SNP pairs were evaluated to detect two QTL signals ($h^2 = 0.1$) based on a multi-SNP model.

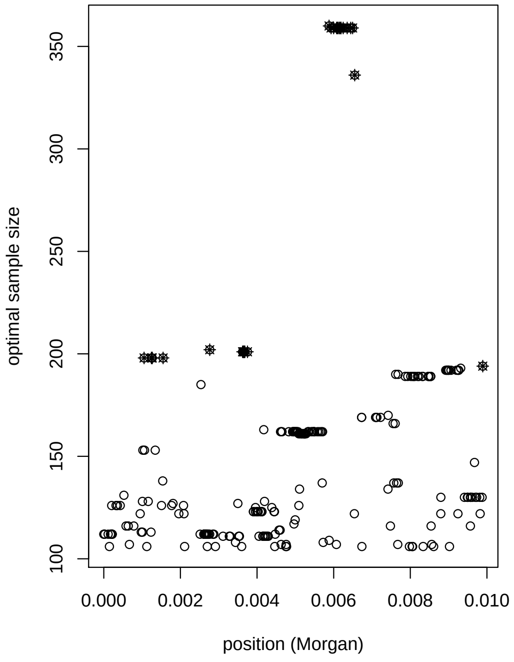

(a)

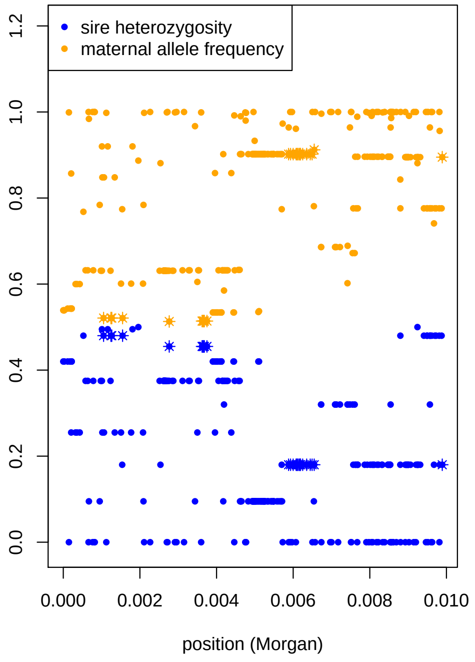

(b)

Figure S.6 Relationship of optimal sample size with genome position. (a) Optimal sample size for detecting one QTL signal was estimated based on the multi-SNP model ($h^2 = 0.1$). All possible SNP positions were evaluated. (b) Sire heterozygosity and maternal allele frequency at each SNP position. Values for SNPs that belong to 10 % highest sample size ($n_{\text{opt}} \geq 194$) are indicated by a star. Results are based on a single simulated data set with $N = 10$ sires.

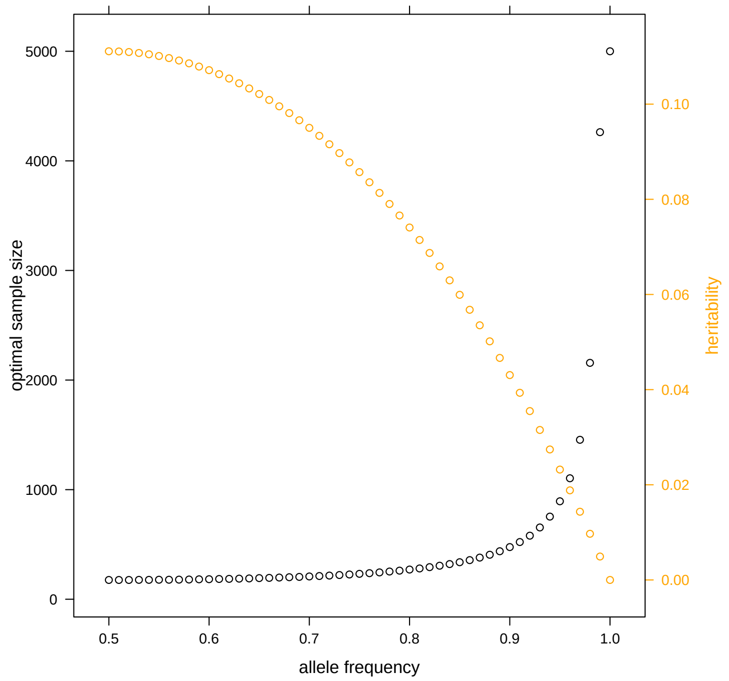

Figure S.7 Dependence of optimal sample size on major allele frequency (p). The relative effect size on the observed genotype level was fixed at 0.5 and multiplied by $\sqrt{2p(1-p)}$. Optimal sample size was estimated based on a single-SNP model.
